# The Association of Neutrophil/Lymphocyte and Platelet/Lymphocyte Ratios and Hematological Parameters with Diagnosis, Stages, Extrapulmonary Involvement, Pulmonary Hypertension, Response to Treatment, and Prognosis in Patients with Sarcoidosis

**DOI:** 10.1155/2020/1696450

**Published:** 2020-09-24

**Authors:** Celalettin Korkmaz, Sinan Demircioglu

**Affiliations:** ^1^Department of Chest Diseases, Meram Faculty of Medicine, Necmettin Erbakan University, Konya, Turkey; ^2^Department of Internal Medicine, Division of Hematology, Meram Faculty of Medicine, Necmettin Erbakan University, Konya, Turkey

## Abstract

Sarcoidosis is a rare disease characterized by granulomatous inflammation in affected organs, primarily in lungs. Neutrophil/lymphocyte ratio (NLR) and platelet/lymphocyte ratio (PLR) are easy and practical methods providing valuable information in diagnosis, severity, and prognosis of various diseases. Here, we aimed to investigate the association between NLR, PLR, and hematological parameters in sarcoidosis. The study was performed with 75 sarcoidosis patients and 92 controls. Patients' NLR, PLR, and hematological parameters were compared with those of controls. Additionally, while differences between NLR and PLR were investigated in sarcoidosis patients, differences of extrapulmonary involvement, pulmonary hypertension (PH), and spontaneous remission between those with and without responses to treatment concerning stages were also assessed. NLR and PLR were significantly higher in sarcoidosis patients than controls. For NLR, sensitivity, specificity, positive predictive value (PPV), and negative predictive value (NPV) were found as 68, 61, 58, and 70% respectively, while sensitivity, specificity, PPV, and NPV for PLR were found as 72, 67, 63, and 74%, respectively. In sarcoidosis patients, NLR and PLR were significantly higher at stage-2 and -3 than at stage -1 and -4. There was a significant weak positive correlation between C-reactive protein (CRP) and NLR and PLR. Mean platelet volume (MPV), hemoglobin (Hgb), and mean corpuscular volume (MCV) were lower among patients than controls. A positive moderate correlation was detected between NLR and CD4/CD8 in blood, while there was a strong positive correlation between CD4/CD8 in bronchoalveolar lavage (BAL) and positive moderate correlation between PLR and CD4/CD8 in BAL. High NLR and PLR values were not significantly associated with pulmonary PH, spontaneous remission, response to treatment, and prognosis. The increase in PLR and NLR may be a guide for diagnoses of both sarcoidosis and lung parenchymal involvement. To use these entities as markers, our findings should be supported with prospective studies with larger samples.

## 1. Introduction

Sarcoidosis is an inflammatory disease of unknown origin and characterized by the formation of granulomas in the affected organs, primarily in the lungs. Patients are at risk of fibrosis in the lungs and irreversible damages to other organs. The condition develops in genetically susceptible individuals and with exposure to an unknown antigen. The accumulation of typical T cells, local T-cell immune response, and granuloma formation in the lungs are the indicatives of the inflammatory response, possibly induced by specific antigens, in sarcoidosis, including autoantigens. Due to its unknown etiology, sarcoidosis has no specific treatment modality and pathognomic markers. Therefore, improved markers are needed to identify disease activity and describe the patients who are at risk of developing fibrosis [1].

As well as radiological and clinical findings, the diagnosis of sarcoidosis is conducted with the histopathological examination of granuloma with epiteloid cell showing no caseification in multiple systems and the exclusion of other reasons leading to granulomatous inflammation [2]. Neutrophils and lymphocytes are important blood cells, involved in the inflammation process. While neutrophils are the first barriers to launch the defensive mechanism in systemic inflammation, lymphocytes constitute the regulatory and protective components of inflammation [3]. Neutrophil-lymphocyte ratio (NLR) is an extremely cost-effective inflammatory parameter to calculate the ratio easily by dividing the absolute neutrophil count by the absolute lymphocyte count from complete blood count. In recent years, NLR has become an easy and practical modality to provide valuable information in the diagnosis and determination of various diseases [4]. NLR was shown to increase in many diseases causing inflammation, such as metabolic syndrome, hypercholesterolemia, diabetes mellitus (DM), hepatic cirrhosis, psoriasis vulgaris, psoriatic arthritis, cardiovascular diseases (CVD), and other malignancies, commonly seen in the community [5–7]. In a study performed by Hammad et al., NLR was stated to be associated with Behcet's disease [8]. In other studies, NLR was also reported to be associated with the severity and prognosis of various respiratory and heart diseases [9, 10]. Higher NLR values in Hodgkin lymphoma patients were found to be significantly associated with disease stage, early-stage risk scoring, and response to treatment [11]. In addition, NLR has been reported to be effective in predicting the disease severity in early stages of coronavirus disease 2019 (COVID-19), the most important challenge at present. In the study by Liu et al., it was stated that patients with NLR ≥ 3.13 and over 50 years of age were prone to severe disease and should have rapid access to the intensive care unit (ICU), if necessary [12]. Another recent study also reported that novel coronavirus can have effects primarily on lymphocytes, especially T lymphocytes, and monitoring the subgroups of NLR and lymphocytes is beneficial in the diagnosis and treatment and the early detection of critical patients with COVID-19 disease [13].

Platelet/lymphocyte ratio (PLR) is a parameter obtained by dividing platelet count by lymphocyte count. In recent years, PLR value has also been reported to be used to indicate inflammation [14]. Publications in recent years have revealed a positive correlation between inflammatory markers and both NLR and PLR. These markers include tumor necrotizing factor-*α* (TNF-*α*) and interleukin-6 (IL-6) in both cardiac and noncardiac patients. In addition, peripheral artery disease and some malignancies have been stated to be associated with high PLR levels [15–18]. On the contrary, PLR was suggested to be associated with rheumatological conditions [19] and cancer [20].

As well as investigating NLR, PLR, mean platelet volume (MPV), C-reactive protein (CRP), erythrocyte sedimentation rate (ESR), and blood and bronchoalveolar lavage (BAL) CD4/CD8 ratios in our study, the association between the features such as the diagnosis, stages, extrapulmonary involvement, pulmonary hypertension (PH), and response to treatment and prognosis has also been assessed in patients with sarcoidosis. We considered that the results found in our study would contribute to whether these parameters can be used as a marker in the early diagnosis and follow-up of sarcoidosis.

## 2. Materials and Methods

Including also a control group, the study was performed with the patients diagnosed in our chest disease department, showing no caseification necrosis along with clinical and radiological findings, and also diagnosed with sarcoidosis by revealing epiteloid-cell granuloma via the histopathological examination and excluding other causes leading to granulomatous inflammation. The study was conducted under the good clinical practice (GCP) and the Helsinki Declaration of 1960 and its later amendments. Approval was obtained from the ethics committee of Meram Medical Faculty (approval number: 220/2427). The records of all sarcoidosis patients over the age of 18 years between 2010 and February 2020 were scanned from the hospital information management system. Based on clinical, radiological, and histopathological investigations, 121 sarcoidosis patients with final diagnosis were determined. Twenty-five and 21 patients were excluded from the study and control groups according to the exclusion criteria and missing follow-up data, respectively. The study group was composed of 75 patients with sarcoidosis, and 92 patients of similar age and gender ratio (male/female) to sarcoidosis patients admitted to the hematology outpatient clinic (based on 762 patients' history and the examination of radiological and laboratory findings) were included into the study as the control group. Such features, including age, gender, posteroanterior (PA) chest radiographs, thoracic computerized tomography (CT), high-resolution lung tomography (HRCT), hemoglobin (Hgb) values in diagnosis, ESR, CRP levels, sarcoidosis stages, echocardiography.(ECHO) results, data of extrapulmonary involvement, consultation reports, and history of the clinical course taken in the outpatient clinic, were recorded. Patients with any metabolic disease, cancer, rheumatological disorder, vasculitis, inflammatory bowel disease, hematological disease, autoimmune disorder, and cardiovascular disease (CVD), or another pulmonary disease, were not included into the study and control groups. Sarcoidosis patients were classified as follows: stage-0, those with normal chest radiology; stage-1, those with hilar lymphadenopathy (LAP); stage-2, those with parenchymal involvement along with hilar LAP; stage-3, those with only parenchymal involvement; and stage-4, those with pulmonary fibrosis [21]. There were only four stage-0 patients among all of the patients diagnosed with sarcoidosis in our department. While two of these four patients were ruled out of the study due to additional diseases, the other two patients were also excluded from the study due to the missing hospital records. For this reason, the study was performed with stage-1, -2, -3, and -4 sarcoidosis patients.

Such parameters as white blood cell (WBC) count, neutrophil, lymphocyte, monocyte, eosinophil, platelet, Hgb, MCV, and MPV values and rates of NLR and PLR between the study and control groups were compared in our study. The correlations between NLR and PLR and CD4/CD8 ratios in blood and BAL were investigated. The difference between NLR and PLR rates of sarcoidosis patients and between the stages of disease was investigated. In addition, whether there was a difference between stage-1 and -4 patients (without radiological findings of alveolitis) and those in stage-2 and stage-3 (with radiological findings of alveolitis) was assessed in terms of NLR and PLR rates. In order to see the difference, NLR and PLR were also investigated between those with and without extrapulmonary involvement, those with and without PH, and those with and without spontaneous remission. Finally, whether NLR and PLR can be used as a marker in these issues was also examined.

### 2.1. Statistical Analyses

For statistical analyses, Statistical Package for Social Sciences, version 22.0 for Windows (SPSS, Chicago, IL, USA), was used to measure the appropriateness for normal distributions. Student's *t*-test was utilized for continuous numerical variables with normal distribution. However, variables without normal distribution were evaluated using the Mann–Whitney *U* test. The receiver operating characteristic (ROC) curve was plotted for the sensitivity and selectivity of NLR and PLR. The correlation between the variables was also evaluated by the Pearson correlation analysis. In all analyses, a value of *p* < 0.05 was considered to be significant.

## 3. Results

While the mean age of sarcoidosis patients was 53.88 ± 13.37, that of our control group was found to be 53.43 ± 11.94 (*p*=0.785). Of patients with sarcoidosis, 16 (21.3%) were male, and 59 (78.7%) were female. Even so, 20 (21.7%) of the control group were male, and 72 (78.3%) were female (*χ*2 = 0.04, *p*=0.949).

In the study group including patients with sarcoidosis, NLR rate (3.26 ± 2.13) was observed to be significantly higher compared with that of the control group (2.45 ± 2.41) (*p* < 0.001). In addition, PLR (192.03 ± 96.76) was found to be significantly higher in the study group compared with the control group (133.99 ± 116.20) (*p* < 0.001) (Table 1).

**Figure 1:**
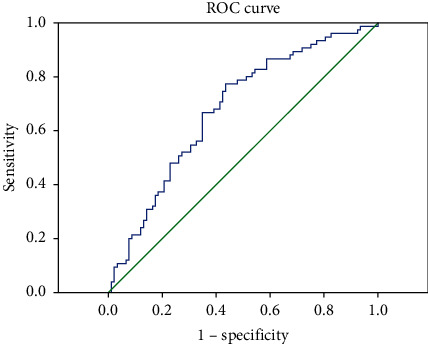
NLR, ROC curve.

**Figure 2:**
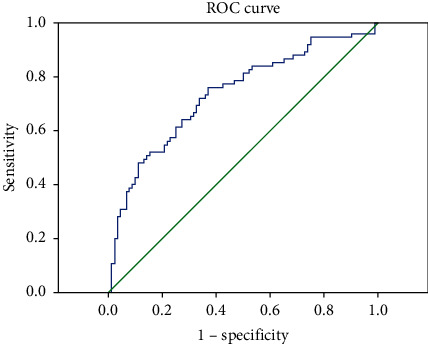
PLR, ROC curve.

The receiver operating characteristic (ROC) curve was plotted for the sensitivity and selectivity of NLR and PLR (Figures 1 and 2). For NLR, the cutoff value was considered as 2.07, and the sensitivity and selectivity rates were found as 68 and 61%, respectively. For PLR, however, the cutoff value was considered as 138.54, and the rates were detected to be 72% for the sensitivity and 67%, for the selectivity. The positive predictive value (PPV) and negative predictive value (NPV) of the NLR category were determined as 58 and 70%, whereas PPV and NPV of the PLR category were 63 and 74%, respectively.

Comparing the hematological parameters between patients with sarcoidosis and the controls, the lymphocyte count was significantly lower in the patient group (1838.67 ± 860.57) than that of the control group (2393.18 ± 796.18) (*p* < 0.001). Even so, Hgb was seen to be significantly lower in the patient group (13.02 ± 1.53) than the controls (13.60 ± 1.16) (*p* < 0.006). While MCV was found lower in the patient group (82.13 ± 7.04) than the control group (85.08 ± 4.38) (*p*=0.001), MPV was also detected to be lower in the patient group (9.59 ± 1.36) compared with that of the control group (10.2 ± 1.32) (*p*=0.04). No significant difference was observed between both groups in terms of leukocyte, neutrophil, monocyte, eosinophil, and platelet counts (*p* > 0.05) (Table 2).

When NLR and PLR were values compared in terms of the stages of sarcoidosis, no significant difference was found between stage-1, -2, -3, and -4 sarcoidosis patients. However, when stage-1 sarcoidosis patients without parenchymal involvement (22 patients with little or no alveolitic phase) and one stage-4 patient with fibrosis in the parenchyma were compared with stage-2 and -3 sarcoidosis patients (46 stage-2 and six stage-3) having many different nonfibrosis involvements in the parenchyma including the prominent alveolitic phase, both NLRs (2.23 ± 0.8 (stage-1 and -4) and 3.72 ± 2.36 (stage-2 and -3), *p*=0.003) and PLRs (149.74 ± 59.57 (stage-1 and -4) and 210.74 ± 104.36 (stage-2 and -3), *p*=0.009) were seen to be significantly lower (Table 3).

In terms of lymphocyte counts, when stage-1 and -4 sarcoidosis patients were compared with those in stage-2 and stage-3 groups, the lymphocyte count in peripheral blood of stage-2 and -3 (alveolitic phase) patients was found to be significantly lower (1/1.69) than that of stage-1 and -4 patients (*p*=0.03) (Table 3).

While mean CRP values of our patients with sarcoidosis were 12.16 ± 15.71, the averages of ESR values were detected as 24.57 ± 18.36.

When the correlation between NLR and CRP was investigated, it was seen that a significant weak positive correlation existed (*r* = 0.31, *p*=0.06) and that there was also a significant weak positive correlation between PLR and CRP (*r* = 0.28, *p*=0.01). Even so, no significant correlation was determined between NLR and ESR (*p*=0.11).

Among all sarcoidosis patients, 38 had the values of CD4/CD8, and the CD4/CD8 value in sarcoidosis patients' blood was found as 1.32 while that in BAL was 3. Considering the correlation between NLR and blood CD4/CD8 and BAL CD4/CD8 ratios, although there was a moderate positive correlation between NLR and blood CD4/CD8 (*r* = 0.558, *p*=0.02), a strong positive correlation was found between NLR and BAL CD4/CD8 ratio (*r* = 0.65, *p*=0.01).

However, even though a moderate positive correlation was detected between PLR and BAL CD4/CD8 ratio (*r* = 0.53, *p*=0.04), there was no significant correlation between PLR and CD4/CD8 ratio in blood.

Forty-six of our sarcoidosis patients showed pulmonary involvement alone, while 29 also had extrapulmonary involvement along with pulmonary involvement. Among 29 extrapulmonary involvements, the affected sites of extrapulmonary manifestations were distributed as follows: nine involvements on skin, seven with cardiac involvement, four with Löfgren's syndrome, three with extrathoracic lymph node involvement, two on eyes, one with parotid gland involvement, one on kidneys, one with neurologic involvement, and one on the rectum.

As to NLR and PLR, no significant difference was found between the patients with pulmonary involvement alone and those with extrapulmonary involvement accompanied by pulmonary involvement and also between the subgroups of those with extrapulmonary involvement (*p*=0.46 and *p*=0.74).

Given the transthoracic ECHO findings, while pulmonary artery systolic pressure (PASP) was found above 25 in 20 (26.7%) patients, PASP was detected as ≤25 in 55 (73.3%) patients. There was no significant difference between these two groups in terms of NLR and PLR (*p*=0.83 and *p*=0.74).

During the follow-up period, spontaneous remission was detected in 27 (36%) of our patients, but not 48 (64%). Additionally, no significant difference was observed between NLR and PLR findings of the groups with and without spontaneous remission (*p*=0.15 and *p*=0.24).

Thirty-three (44%) patients were determined to receive no treatment. Of 33 untreated patients, 27 had spontaneous remission, while six without spontaneous remission would be treated according to their follow-up, if progression developed. Even so, 42 (56%) of our patients were found to undergo a treatment modality with one of the following agents: methylprednisolone, methotrexate, infliximab, local steroids, or nonsteroidal anti-inflammatory drugs (NSAIDs). There was also no significant difference between NLR and PLR of patients receiving and not receiving treatment (*p*=0.12 and *P*=0.19).

A progression was observed in three of 42 patients receiving treatment. There was no significant difference between NLR and PLR findings of those responding to the treatment and those showing no improvements (*p*=0.39 and *p*=0.51).

When our patients were evaluated according to their latest health status, it was observed that 48 (64%) patients had regression, and 24 (32%) patients were stable, while there were progressions only in three (4%) patients. There was no significant difference between NLR and PLR findings of these groups (*p*=0.60 and *p*=0.13).

## 4. Discussion

In the present study, NLR was found to be significantly higher in the patient group with sarcoidosis compared to the controls. The cutoff value was taken as 2.07 for NLR, and the sensitivity and specificity rates were found as 68 and 61%, while the rates of PPV and NPV were detected as 58 and 70%, respectively. Additionally, PLR was also found to be significantly higher in the patients with sarcoidosis compared to the controls, and when the cutoff value was taken as 138.54 for PLR, the rates of sensitivity, selectivity, PPV, and NPV were determined as 72, 67, 63, and 74%, respectively. In one of the previous studies performed by Dirican et al., NLR was stated to be significantly higher in patients with sarcoidosis than that in healthy controls. In the same study, the cutoff value of NLR was calculated as 2, and according to the cutoff value, the sensitivity and specificity values were found to be 80 and 59%, respectively. In light of such findings, it was suggested that NLR can be a sensitive biomarker and can be utilized in clinical practice to define patients' prognosis [22]. The findings in our study are also compatible with those reported in the study by Dirican et al.

In the study performed by Ocal et al., a significant difference was found between mean NLR and ESR and the radiological stages performed according to the chest radiograph and the total high-resolution computed tomography (HRCT) score (THS) (based on the parenchymal involvement). In the same study, statistically significant positive correlations were detected between NLR and WBC, NLR and THS, NLR and ESH, THS and ESR, ESR and platelets, WBC and neutrophils, and WBC and lymphocytes. Based on these findings, it was suggested that NLR could be used as a prognostic marker in sarcoidosis [23]. No correlation was found between NLR and ESR in our study, but there was a significant weak positive correlation between NLR and CRP. The rates of NLR showed no difference between radiological stages, when compared one by one. However, when stage-2 and -3, where the alveolitic phase was predominant, were compared with stage-1 and -4, where no radiological parenchymal involvement was present, but there was fibrosis, NLR rate was detected to be significantly higher (3.72 ± 2.36 and 2.23 ± 0.8) because there was the migration of lymphocytes from peripheral blood to lung parenchyma during stage-2 and -3 and perhaps because of increased inflammation within this period. When the two groups were compared in terms of lymphocyte numbers, it was found that the lymphocyte count in peripheral blood of stage-2 and -3 patients was significantly lower (1/1.69) than that of stage-1 and -4 patients. In the 1960s and early 1970s, the observation of lymphopenia and cutaneous anergy in peripheral blood (delayed hypersensitivity to antigens such as purified protein derivative (PPD) and lack of skin reactions) suggested that sarcoidosis was due to the deficiency of T cells. However, the advent of flexible bronchoscopy and BAL in the late 1970s led to the discovery of enlarged lymphocyte populations in the lungs and affected tissues of patients with extrapulmonary involvement, on a large scale. The total number of cells and lymphocytes in the BAL fluid was significantly higher in patients with parenchymal infiltration. It was reported by subsequent studies that such an expanded lymphocyte population included CD4-positive (CD4+) T-helper cells, which could transform predominantly into TH1 and differ to TH17 effector cells producing IL-17 and TH17.1 cells producing IFN-*γ* [24–26]. In our study, CD4/CD8 ratios in blood and BAL were found to be 1.32 and 3 in patients with sarcoidosis, respectively. When the correlation between NLR and blood CD4/CD8 ratio was investigated, a moderate positive correlation was found. However, a strong positive correlation was seen between NLR and BAL CD4/CD8.

In the study by Ozdemir et al., both NLR and PLR were found significantly higher among patients with sarcoidosis than those in the control group [27]. In a study investigating the correlation between these parameters and inflammation in patients with end-stage renal disease (ESRD), PLR was shown to be a superior inflammatory marker to NLR [28]. Also, in our study, PLR was significantly higher in patients with sarcoidosis than that of the control group. In addition, PLR was significantly higher in stage-2 and -3 patients (210.74 ± 104.36 and 149.74 ± 59.57) than that of stage-1 and -4 patients due to lymphocyte migration from peripheral blood to lung parenchyma and perhaps increased inflammation developing within stage-2 and -3. There was a moderate significant correlation between PLR and BAL CD4/CD8 ratio in bronchoalveolar lavage. However, no significant correlation was observed between PLR and CD4/CD8 ratio in blood. A significant, weak positive correlation was also seen between PLR and CRP.

PH is a fearful complication in sarcoidosis patients as an important negative prognostic factor for lung transplantation that may be required in the advanced stage [29]. PH is considered to be a vasculopathy caused by excessive vascular cell growth, along with inflammation, playing the major role in the disease process [30]. Previous studies revealed that NLR was significantly higher in PH patients compared to healthy volunteers [31] and reported that NLR could be useful in assessing disease severity [32]. In another study performed by Mirsaeidi et al., it was concluded that the level of inflammation may be related to the development of PH in sarcoidosis patients. In addition, it was concluded that NLR is not an ideal screening tool because of its low sensitivity and PPV rates for diagnosing PH in sarcoidosis patients, but NLR may be a good negative predictive test that is widely available in a simple, inexpensive, and office-based environment in order to estimate the PH risk in sarcoidosis patients due to its relatively good specificity (81.4%) [33]. In our study, when the patients with pulmonary arterial pressure (PAP) > 25 through transthoracic echocardiography (ECHO) and those with PAP ≤ 25 were compared, no significant difference was found, and the difference may be due to the fact that PH was not confirmed by the right heart catheterization in both our study and the study by Mirsaeidi et al. This issue is also one of the important limitations in our study. Sarcoidosis-related pulmonary hypertension (SRPH) is encountered in 5 to 20% of sarcoidosis patients. Increased PAP may be due to comorbidities such as cardiac sarcoidosis and sleep apnea, as well as many reasons, such as vasculocentric, parenchymal, and mechanical factors. Most SRPH patients have fibrotic lung disease, but SRPH may also be witnessed among those without advanced parenchymal lung disease. ECHO still remains an important tool for the evaluation of SRPH but can reveal the severity of PAP, both higher and lower. So, right heart catheterization remains being the definitive test to diagnose the condition [34].

Apart from hemostasis, platelets are of vital importance in angiogenesis, inflammation, allergic reactions, the repair and regeneration of tissues, and in the release of chemokines and cytokines, playing a part in the production of a strong response [35, 36]. Many studies have asserted findings referring to the correlation between high MPV values and active inflammatory disease [37]. In a few small-size studies performed in patients with rheumatoid arthritis, an association was revealed to be present between high MPV and increased disease activity and inflammatory markers [38, 39]. Additionally, a similar correlation has been shown in the decrease in MPV after the treatment among patients with ankylosing spondylitis [40]. In the aforementioned studies, MPV was reported to be higher between patients with rheumatoid arthritis and ankylosing spondylitis than the controls [41, 42]. However, in another study, the findings related to rheumatoid arthritis and ankylosing spondylitis were contradictory, and while MPV was reported to be lower in those with active disease, an increase was seen in MPV among patients receiving rheumatoid arthritis treatment [37]. In our study, MPV was significantly lower in the study group (9.59 ± 1.36) than the controls (10.02 ± 1.32). However, platelet count was higher in patients with sarcoidosis (294,600 ± 79,786 and 274,586 ± 76,176), but the difference was not statistically significant.

Anemia and leukopenia can be encountered in patients with sarcoidosis. It was considered that leukopenia may also be due to the accumulation and redistribution of T cells in active inflammation sites, as well as hypersplenism or bone marrow involvement, and leukemoid reaction, eosinophilia, and thrombocytopenia are not commonly seen [43, 44]. In our study, Hgb was significantly lower in the sarcoidosis group (13.02 ± 1.53) than the control group (13.60 ± 1.16). Furthermore, MCV was also found to be lower in the study group (82.13 ± 7.04) than the controls (85.08 ± 4.38).

Spontaneous remission was detected in 27 (36%) of our patients, but not 48 (64%) subjects. There was no significant difference between NLR and PLR rates of the patients with and without spontaneous remission. Of our 75 patients, 42 (56%) were determined to be treated with agents such as methylprednisolone, methotrexate, infliximab, local steroids, or NSAIDs, while 33 (44%) received no treatment. Among 33 untreated patients, 27 had spontaneous remission, while six without spontaneous remission would be treated according to their follow-up. There was no significant difference between NLR and PLR of patients receiving and not receiving treatment.

Based on our patients' latest assessments, it was observed that 48 (64%) patients had regression, 24 (32%) were stable, and 3 (4%) patients developed progression. In terms of NLR and PLR, no significant difference was observed between these groups.

### 4.1. Limitations of the Study

The biggest limitation of our study is that our study is a retrospective study; therefore, there are no CRP, ESR, ECO, and CD4/CD8 values in the control group. CD4/CD8 ratio measurements are available in 38 of our sarcoidosis patients but not all of them. One of the important limitations of our study was that PH was not confirmed by right heart catheterization.

## 5. Conclusion

In conclusion, NLR and PLR are inexpensive and easily accessible parameters. Therefore, we consider that the increase in PLR and NLR values may be a guide both for the diagnosis of sarcoidosis and lung parenchyma involvement and that our findings should be supported by prospective studies with larger samples in order to elucidate the use of these entities in prognosis.

## Figures and Tables

**Table 1:** The comparison of NLR and PLR between the study and control groups.

	Patients with sarcoidosis, mean ± SD (*n* = 75)	Controls, mean ± SD (*n* = 92)	*p*
NLR	3.26 ± 2.13	2.45 ± 2.41	<0.001
PLR	192.03 ± 96.76	133.99 ± 116.20	<0.001

NLR: neutrophil/lymphocyte ratio, PLR: platelet/lymphocyte ratio, and SD: standard deviation.

**Table 2:** The comparison of hematological parameters between the study and control groups.

	Patients, mean ± SD (*n* = 75)	Controls, mean ± SD (*n* = 92)	*p*
Leukocyte (cells/mm^3^)	7684.53 ± 2497.24	7914.45 ± 1990.35	0.509
Neutrophils (cells/mm^3^)	4971.20 ± 2124.93	4760.97 ± 1843.58	0.495
Lymphocytes (cells/mm^3^)	1838.66 ± 860.57	2393.18 ± 796.18	<0.001
Monocytes (cells/mm^3^)	608.13 ± 271.52	550.77 ± 165.72	0.095
Eosinophils (cells/mm^3^)	184.93 ± 168.09	159.79 ± 127.85	0.274
Hgb (g/dL)	13.02 ± 1.53	13.60 ± 1.16	0.006
MCV (fL)	82.13 ± 7.04	85.08 ± 4.38	0.001
Platelets (cells/mm^3^)	294600.00 ± 79786.53	274586.95 ± 76176.22	0.100
MPV (fL)	9.59 ± 1.36	10.02 ± 1.32	0.044

Hgb: hemoglobin, MCV: mean corpuscular volume, MCV: mean platelet volume, and SD: standard deviation.

**Table 3:** The comparison of NLR and PLR between sarcoidosis patients at stage-1 and -4 and stage-2 and -3.

	Stage-1 and -4, mean ± SD (*n* = 23)	Stage-2 and -3, mean ± SD (*n* = 52)	*p*
NLR	2.23 ± 0.85	3.72 ± 2.36	0.003
PLR	149.74 ± 59.57	210.74 ± 104.36	0.009

NLR: neutrophil/lymphocyte ratio, PLR: platelet/lymphocyte ratio, and SD: standard deviation.

## Data Availability

The data used to support the findings of this study are available from the corresponding author upon request.

## References

[1] Grunewald J. (2019). Sarcoidosis. *Nature Reviews Disease Primers*.

[2] Zissel G., Müller-Quernheim J. (1998). Sarcoidosis: historical perspective and immunopathogenesis (part I). *Respiratory Medicine*.

[3] Rocha‐Pereira P., Santos-Silva A., Rebelo I., Figueiredo A., Quintanilha A., Teixeira F. (2004). The inflammatory response in mild and in severe psoriasis. *British Journal of Dermatology*.

[4] Demirkol S., Balta S., Kucuk U., Kucuk H. O. (2014). The neutrophil lymphocyte ratio may be useful inflammatory indicator before applying other expensive and invasive procedures. *Indian Journal of Nephrology*.

[5] An I., Ucmak D. (2018). Evaluation of neutrophil-to-lymphocyte ratio, platelet-to-lymphocyte ratio, mean platelet volume, and C-reactive protein in patients with psoriasis vulgaris. *Dicle Medical Journal*.

[6] Balta S., Celik T., Mikhailidis D. P. (2016). The relation between atherosclerosis and the neutrophil-lymphocyte ratio. *Clinical and Applied Thrombosis/hemostasis*.

[7] Peng Y., Li Y., He Y. (2018). The role of neutrophil to lymphocyte ratio for the assessment of liver fibrosis and cirrhosis: a systematic review. *Expert Review of Gastroenterology & Hepatology*.

[8] Hammad M., Shehata O. Z., Abdel-Latif S. M., El-Din A. M. M. (2018). Neutrophil/lymphocyte ratio and platelet/lymphocyte ratio in Behçet’s disease: which and when to use?. *Clinical Rheumatology*.

[9] Uthamalingam S., Patvardhan E. A., Subramanian S. (2011). Utility of the neutrophil to lymphocyte ratio in predicting long-term outcomes in acute decompensated heart failure. *The American Journal of Cardiology*.

[10] Kayrak M., Erdoğan H. İ., Solak Y. (2014). Prognostic value of neutrophil to lymphocyte ratio in patients with acute pulmonary embolism: a restrospective study. *Heart, Lung and Circulation*.

[11] Dogan A., Demircioglu S. (2019). Assessment of the neutrophil-lymphocyte ratio in classic Hodgkin lymphoma patients. *Pakistan Journal of Medical Sciences*.

[12] Liu J., Liu Y., Xiang P. (2020). Neutrophil-to-lymphocyte ratio predicts severei llness patients with 2019 novel coronavirus in the early stage. *MedRxiv*.

[13] Qin C., Zhou L., Hu Z. (2020). Dysregulation of immune response in patients with coronavirus 2019 (COVID-19) in Wuhan, China. *Clinical Infectious Diseases*.

[14] Zhang J., Zhang H.-Y., Li J., Shao X.-Y., Zhang C.-X. (2017). The elevated NLR, PLR and PLT may predict the prognosis of patients with colorectal cancer: a systematic review and meta-analysis. *Oncotarget*.

[15] Wang D., Yang J. X., Cao D. Y. (2013). Preoperative neutrophil-lymphocyte and platelet-lymphocyte ratios as independent predictors of cervical stromal involvement in surgically treated endometrioid adenocarcinoma. *OncoTargets and Therapy*.

[16] Gary T., Pichler M., Belaj K. (2013). Platelet-to-lymphocyte ratio: a novel marker for critical limb ischemia in peripheral arterial occlusive disease patients. *PLoS One*.

[17] Azab B., Shah N., Akerman M., McGinn J. T. (2012). Value of platelet/lymphocyte ratio as a predictor of all-cause mortality after non-ST-elevation myocardial infarction. *Journal of Thrombosis and Thrombolysis*.

[18] Raungkaewmanee S., Tangjitgamol S., Manusirivithaya S., Srijaipracharoen S., Thavaramara T. (2012). Platelet to lymphocyte ratio as a prognostic factor for epithelial ovarian cancer. *Journal of Gynecologic Oncology*.

[19] Gasparyan A. Y., Ayvazyan L., Mukanova U., Yessirkepov M., Kitas G. D. (2019). The platelet-to-lymphocyte ratio as an inflammatory marker in rheumatic diseases. *Annals of Laboratory Medicine*.

[20] Li B., Zhou P., Liu Y. (2018). Platelet-to-lymphocyte ratio in advanced cancer: review and meta-analysis. *Clinica Chimica Acta*.

[21] Greco F., Spagnolo P., Muri M. (2014). The value of chest radiograph and computed tomography in pulmonary sarcoidosis. *Sarcoidosis, Vasculitis, and Diffuse Lung Diseases: Official Journal of WASOG*.

[22] Dirican N., Anar C., Kaya S., Bircan H. A., Colar H. H., Cakir M. (2016). The clinical significance of hematologic parameters in patients with sarcoidosis. *The Clinical Respiratory Journal*.

[23] Ocal N., Dogan D., Ocal R. (2016). C101 sarcoidosis: relation of neutrophil/lymphocyte ratio with radiological extent in pulmonary sarcoidosis. *American Journal of Respiratory and Critical Care Medicine*.

[24] Miyoshi S., Hamada H., Kadowaki T. (2010). Comparative evaluation of serum markers in pulmonary sarcoidosis. *Chest*.

[25] Facco M., Cabrelle A., Teramo A. (2011). Sarcoidosis is a Th1/Th17 multisystem disorder. *Thorax*.

[26] Broos C. E., Koth L. L., Van Nimwegen M. (2018). Increased T-helper 17.1 cells in sarcoidosis mediastinal lymph nodes. *European Respiratory Journal*.

[27] Ozdemir C., Sökücü S., Önür S. (2018). Can neutrophil/lymphocyte ratio and platelet/lymphocyte ratio be used in differential diagnosis of stage I sarcoidosis from tuberculosis lymphadenopathy?. *Eurasian Journal of Pulmonology*.

[28] Turkmen K., Erdur F. M., Ozcicek F. (2013). Platelet-to-lymphocyte ratio better predicts inflammation than neutrophil-to-lymphocyte ratio in end-stage renal disease patients. *Hemodialysis International*.

[29] Baughman R. P., Engel P. J., Meyer C. A., Barrett A. B., Lower E. E. (2006). Pulmonary hypertension in sarcoidosis. *Sarcoidosis Vasculitis and Diffuse Lung Diseases*.

[30] Baughman R. P. (2007). Pulmonary hypertension associated with sarcoidosis. *Arthritis Research & Therapy*.

[31] Yıldız A., Kaya H., Ertaş F. (2013). Association between neutrophil to lymphocyte ratio and pulmonary arterial hypertension. *Turk Kardiyoloji Dernegi Arsivis: Turk Kardiyoloji Derneginin Yayin Organidir*.

[32] Özpelit E., Akdeniz B., Özpelit M. E. (2015). Prognostic value of neutrophil-to-lymphocyte ratio in pulmonary arterial hypertension. *Journal of International Medical Research*.

[33] Mirsaeidi M., Mortaz E, Omar H. R, Camporesi E. M, Sweiss N (2016). Association of neutrophil to lymphocyte ratio and pulmonary hypertension in sarcoidosis patients. *Tanaffos*.

[34] Shlobin O. A., Baughman R. P. Sarcoidosis-associated pulmonary hypertension.

[35] Ataseven A., Ugur Bilgin A. (2014). Effects of isotretinoin on the platelet counts and the mean platelet volume in patients with acne vulgaris. *The Scientific World Journal*.

[36] Saleh H. M. A., Attia E. A. S., Onsy A. M., Saad A. A., Abd Ellah M. M. M. (2013). Platelet activation: a link between psoriasisper seand subclinical atherosclerosis—a case-control study. *British Journal of Dermatology*.

[37] Leader A., Pereg D., Lishner M. (2012). Are platelet volume indices of clinical use? A multidisciplinary review. *Annals of Medicine*.

[38] Milovanovic M., Nilsson E., Järemo P. (2004). Relationships between platelets and inflammatory markers in rheumatoid arthritis. *Clinica Chimica Acta*.

[39] Yazici S., Yazici M., Erer B. (2010). The platelet indices in patients with rheumatoid arthritis: mean platelet volume reflects disease activity. *Platelets*.

[40] Yazici S., Yazici M., Erer B. (2010). The platelet functions in patients with ankylosing spondylitis: anti-TNF-*α*therapy decreases the mean platelet volume and platelet mass. *Platelets*.

[41] Kim D.-A., Kim T.-Y. (2011). Controversies over the interpretation of changes of mean platelet volume in rheumatoid arthritis. *Platelets*.

[42] Kisacik B., Tufan A., Kalyoncu U. (2008). Mean platelet volume (MPV) as an inflammatory marker in ankylosing spondylitis and rheumatoid arthritis. *Joint Bone Spine*.

[43] Iannuzzi M. C., Rybicki B. A., Teirstein A. S. (2007). Sarcoidosis. *New England Journal of Medicine*.

[44] Bain B. J. (2001). Bone marrow trephine biopsy. *Journal of Clinical Pathology*.

